# Conditionally Reprogrammed Normal and Transformed Mouse Mammary Epithelial Cells Display a Progenitor-Cell–Like Phenotype

**DOI:** 10.1371/journal.pone.0097666

**Published:** 2014-05-15

**Authors:** Francisco R. Saenz, Virginie Ory, Maram AlOtaiby, Sonia Rosenfield, Mary Furlong, Luciane R. Cavalli, Michael D. Johnson, Xuefeng Liu, Richard Schlegel, Anton Wellstein, Anna T. Riegel

**Affiliations:** 1 Department of Oncology, Lombardi Comprehensive Cancer Center, Georgetown University, Washington, District of Columbia, United States of America; 2 Department of Pathology, Lombardi Comprehensive Cancer Center, Georgetown University, Washington, District of Columbia, United States of America; University of Miami School of Medicine, United States of America

## Abstract

Mammary epithelial (ME) cells cultured under conventional conditions senesce after several passages. Here, we demonstrate that mouse ME cells isolated from normal mammary glands or from mouse mammary tumor virus (MMTV)-*Neu*–induced mammary tumors, can be cultured indefinitely as conditionally reprogrammed cells (CRCs) on irradiated fibroblasts in the presence of the Rho kinase inhibitor Y-27632. Cell surface progenitor-associated markers are rapidly induced in normal mouse ME-CRCs relative to ME cells. However, the expression of certain mammary progenitor subpopulations, such as CD49f^+^ ESA^+^ CD44^+^, drops significantly in later passages. Nevertheless, mouse ME-CRCs grown in a three-dimensional extracellular matrix gave rise to mammary acinar structures. ME-CRCs isolated from MMTV-*Neu* transgenic mouse mammary tumors express high levels of HER2/*neu*, as well as tumor-initiating cell markers, such as CD44^+^, CD49f^+^, and ESA^+^ (EpCam). These patterns of expression are sustained in later CRC passages. Early and late passage ME-CRCs from MMTV-*Neu* tumors that were implanted in the mammary fat pads of syngeneic or nude mice developed vascular tumors that metastasized within 6 weeks of transplantation. Importantly, the histopathology of these tumors was indistinguishable from that of the parental tumors that develop in the MMTV-*Neu* mice. Application of the CRC system to mouse mammary epithelial cells provides an attractive model system to study the genetics and phenotype of normal and transformed mouse epithelium in a defined culture environment and *in vivo* transplant studies.

## Introduction

Studies utilizing primary normal and tumor epithelial cells are frequently hampered by the fact that cells can only be cultured for short periods of time before they cease proliferating and undergo senescence [1]. In addition, the cultured cells frequently do not retain lineage commitment or normal proliferation or differentiation potential. Various methods have been used to immortalize epithelial cells, such as introduction of viral oncogenes and the telomerase reverse transcriptase [2], although these interventions frequently disrupt normal differentiation. It has been recently demonstrated that human epithelial cells from a variety of sources (e.g., keratinocytes and human mammary epithelial cells) can be cultured indefinitely and can bypass senescence when cultured on irradiated fibroblast feeders in the presence of the Rho kinase inhibitor Y-27632 [3]. Cells passaged in this system are known as conditionally reprogrammed cells (CRCs). The CRC system has been applied to epithelial cells from human tumor tissues, where drug responsiveness *in vivo* can be predicted from the *in vitro* responses of the CRCs [4]. Thus, the CRC system has potential for studying normal and tumor cells from primary sources in culture without utilizing overexpression of oncogenes and cell cycle inhibitory factors. Further analysis of human CRCs revealed that induction of the CRC phenotype is rapid and involves reprogramming of most of the cell population [5]. However, the CRC phenotype in keratinocytes can be reversed by the removal of the Rho kinase inhibitor and cells can then differentiate normally, as demonstrated by the ability of tracheal epithelium to form a stratified epithelium in a three-dimensional culture system [5]. Of note is that human CRCs share many properties of adult stem cells but do not express markers of pluripotent progenitors [5]. Thus, human CRCs can be used for *in vitro* and *in vivo* studies of normal and tumor cells and may offer a system where drug therapies can be tested on cells expanded from individual patients.

In the current study, we wished to determine if mouse mammary epithelial (ME)-CRCs could be developed from normal or tumor sources, and if their properties mirrored those of human cells exposed to the CRC system. Although mouse epithelial cells undergo senescence with serial passage, the mechanisms of senescence differ from those of human cells [6]. In particular, telomere shortening does not play a major role in driving senescence of mouse cells [7], [8]. Interestingly, despite these differences, we report that both normal and tumor ME-CRCs from mice can be passaged indefinitely. Similar to human epithelial cells, normal mouse ME-CRCs expressed progenitor-associated markers, but not pluripotent stem cell markers. ME-CRCs were able to form mammary acinar structures when grown in a three-dimensional (3D) Matrigel matrix. However, unlike human cells, high expression levels of many progenitor cell markers were maintained after CRC withdrawal, suggesting that, in mouse cells, many of the effects of the CRC system are not rapidly reversible. ME-CRCs derived from mouse mammary tumors dissected from MMTV-*Neu* mice could also be passaged indefinitely, and a large portion of the cells expressed markers characteristic of tumor-initiating cells *in vitro*. Large tumors developed in the mammary fat pad after implantation of these cells. Thus, the CRC system can also be applied to normal and transformed mouse epithelial cells and presents opportunities to study properties of genetically manipulated cells in allograft models.

## Materials and Methods

### Cell culture

Normal and tumor mouse ME cells were isolated from mammary glands 3 and 4 of 6-week-old female FVB or FVB.Cg-Tg(ACTB-EGFP)B5Nagy/J mice (The Jackson Laboratory, Bar Harbor, ME, www.jax.org) and from mammary tumors of MMTV-*Neu* mice, respectively, as described previously [9]. ME-CRCs were maintained on irradiated 3T3-J2 fibroblasts as described previously [3] and passaged in Dulbecco's modified Eagle medium (DMEM)/F12 containing 10 mM Y-27632 (Reagents Direct, Encinitas, CA, www.reagentsdirect.com). Co-culture flasks were trypsinized in two steps by using 0.05% Trypsin-EDTA. The initial 1–2 minutes trypsinization to remove feeders was followed by a wash using phosphate-buffered saline (PBS) and another 5 minutes to detached epithelial cells that were subsequently reseeded at a 1∶10 ratio in the CRC system and cultured for 5–6 days before passaging. Freshly isolated primary cells were defined as P0. Subsequent passages of the ME cells (CRCs or non-CRCs) were referred to as P1 and greater. ME-CRCs early passage was defined as P<10 and late passage as P≥10.

### Array comparative genomic hybridization (cgh) analysis

Normal ME-CRCs (P5, P18, and P76) and MMTV-*Neu* ME-CRCs (P6, P38, and P73), as well as normal ME non-CRCs (P2) and MMTV-HER2/*neu* ME non-CRCs (P2) were analyzed for DNA copy number changes using the oligonucleotide-based 4×180K mouse array CGH platform (Agilent Technologies, Santa Clara, CA, www.agilent.com). Genomic DNA from CRC and non-CRC cultures was isolated and enzymatically digested according to the manufacturer's protocol (Agilent Technologies). Normal mouse DNA, isolated from female mouse tail, was used as reference DNA. Equal amounts of CRC or non-CRC genomic DNA and reference DNA were directly labeled with Cy3 or Cy5, respectively, using the SureTag Labeling Kit (Agilent Technologies) and hybridized in the presence of mouse Cot1-DNA (Life Technologies, Carlsbad, CA, www.lifetechnologies.com) to the arrays for 40 hours. The arrays were scanned using an Agilent array scanner and the data were analyzed using Feature Extraction (FE) software v10.10 and Genome Workbench version 7.0 software (Agilent Technologies). For each sample, FE gave a log_10_ ratio (log of tumor processed signal over reference processed signal for each gene) that was imported into Genome Workbench and transformed and viewed as a log_2_-based ratio. Outliers detected by FE were excluded from the analysis. The algorithm ADM-2 and a threshold value of 6.0 were applied with the appropriate filters to analyze the data. Gene amplifications and deletions were considered when the corresponding plotted oligonucleotide probes presented values of log_2_ >7/6 and <5/6, respectively.

### Flow cytometry

CRCs were analyzed for expression of individual (monoparametric) or multiple (multiparametric) surface proteins using fluorescence-labeled primary antibodies. Sca1/FITC anti-mouse (Cat #553335), CD24/PE anti-mouse (Cat #553262), Rat IgG-FITC anti-mouse (Cat #553929), and Rat IgG-PE (Cat #553989) were all purchased from BD Biosciences (San Jose, CA, www.bdbiosciences.com). Additionally, CD29/APC anti-mouse/rat (Cat #102215), ESA/FITC anti-mouse (Cat #118207), CD49f/APC anti-human/mouse (Cat # 313615) and CD44/PE anti-mouse (Cat #103007) antibodies were purchased from BioLegend (San Diego, CA, www.biolegend.com). In short, samples were washed once with 3% bovine serum albumin (BSA), a second time with PBS and then incubated with a viability assay kit (Life Technologies Cat #L34955) according to manufacturer's instructions. The excess reagent was removed by washing with PBS, then incubated with labeled single antibody or antibody cocktail in PBS at 4° C for one hour. Excess antibody was removed by washing with PBS prior to analysis. Samples were analyzed using a BD FACS Aria Cell Sorter (BD Biosciences). Gating was used to restrict analysis to cells labeled for single or multiple markers, and graphs were plotted using FCS Express 4 software.

### Brightfield and Confocal Microscopic Analysis of Cultures Growing in 3D Matrigel Matrices

ME-CRCs and MCF10A cultures were established in extracellular matrix as previously described [10], [11]. Briefly, 40 µL of cold Matrigel (BD Bioscience Cat #354230) was added to the surface of sterile pre-chilled, slide chambers (Lab-Tek II Chamber Slide Cat #154534) purchased from ThermoFisher Scientific Inc. (Waltham, MA, www.fishersci.com). The slides were then incubated at 37°C for 15 minutes to allow polymerization of the substratum. Subsequently, 3×10^4^ cells/mL were added to the slides and resuspended in assay media containing 2% FBS, 2% Matrigel, and 20 ng/mL EGF and incubated at 37°C until sphere formation. For confocal microscopy, immunostaining of the acinar structures was carried out as described [12].

### Western blotting

Whole-cell extracts were prepared from ME-CRCs, and immunoblot analysis for proteins indicated was performed as described previously [13].

### Allograft analysis

MMTV-*Neu* ME-CRCs were implanted in mammary fat pads of nude or syngeneic FVB mice (The Jackson Laboratory). Briefly, single-cell suspensions of early (P6) and late (P68) passage MMTV-*Neu* ME-CRCs were injected orthotopically, with Matrigel, into mammary gland 4 of 3-month-old MMTV-*Neu* female mice. Mice were euthanized and tumors were removed 6 weeks later or when the longest axis of the tumor reached 1 cm as described previously [14]. Lung and liver tissue was collected at the same time as the primary mammary tumor. Real-time polymerase chain reaction (qRT PCR) for β-casein expression in lung and liver tissues was performed as previously described [15].

### Histological analysis

Hematoxylin and eosin (H&E) staining and immunohistochemical and immunofluorescence analyses as indicated were performed on paraffin-embedded 5- µm sections of mammary gland, lung or liver using standard protocols described elsewhere [14].

### Ethics statement

All procedures using animals were approved by the Georgetown University IACUC and were conducted according to the NIH guidelines for the care and use of laboratory animals (Public Health Service: Assurance # A-3282-01). Isoflurane was used for all animals undergoing surgery.

### Statistical and Image Analyses

Samples were compared using two-tailed Student's *t*-test. P<0.05 was considered statistically significant. Phase contrast images of serially passaged CRCs, micrographs showing immunohistochemical staining of 3D mammosphere cultures, and images of western blots were adjusted for brightness/contrast using Adobe Photoshop CS5 software. All images were adjusted to the same level. ImageJ was used to quantify colony subtypes. Prism 5 for PC (GraphPad, San Diego, CA, www.graphpad.com) was used for all statistical analyses. Figures were assembled using Adobe Illustrator CS5 software.

## Results

### ME-CRCs Maintain a Phenotype Similar to the Parental ME Cells

**Figure 1:**
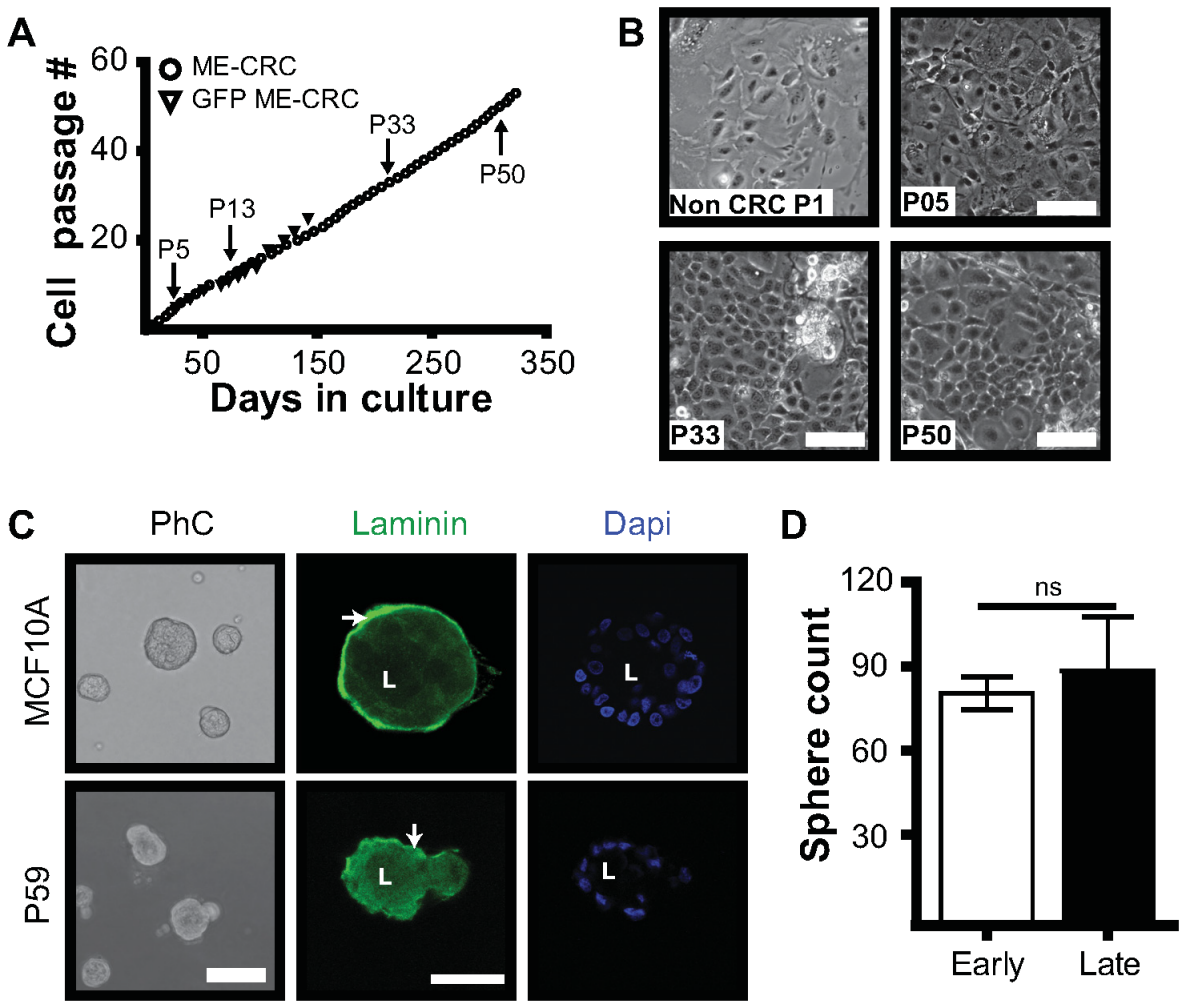
Culture of normal mouse mammary epithelial (ME) cells under conditions generating conditionally reprogrammed cells (CRCs). **A**) Continuous culture of ME-CRCs generated from mammary glands of FVB or FVB-green fluorescent protein (GFP) mice. **B**) Phase contrast images of ME-CRCs and non-CRCs at indicated passages; magnification, 10×; bar, 100 µm. **C**) Phase contrast and immunofluorescence images of mammary acinar-like structures from P59 ME-CRCs and immortalized MCF10A cells stained for laminin and counterstained with DAPI. L, lumen; arrow, peripheral laminin. Magnification, 10× and 63×; bar, 100 µm and 50 µm for phase contrast and fluorescence, respectively. **D**) Average number of spheres per field of early and late ME-CRCs in four independent fields from four wells. Data are representative of two independent experiments. Values are mean±SEM; ns, not significant. **A–D**) All data were acquired after 5 days of culture. P, passage.

We have reported that human epithelial cells can be propagated indefinitely when co- cultured with irradiated fibroblast feeder cells in the presence of the ROCK inhibitor Y-27632 [3]. To determine if mouse ME cells can be similarly re-programmed, we prepared ME cells from mammary glands of FVB mice. When grown on plastic, these cells senesce after 3–4 passages (Figure S1A). However, when grown on the irradiated fibroblasts in the presence of Y-27632 as described [3], [16], the mouse ME cells maintained a normal cobblestone-like morphology in the CRC system for >50 passages after one year in culture (Figure 1A and B). This was also demonstrated with a ME preparation from transgenic FVB mice that harbored a germ line insertion of the green fluorescent protein (GFP) under the control of the actin promoter. This strain assumed similar morphology and could also be grown indefinitely in CRC culture (Figure 1A). To date we have maintained these cells in culture for up to 150 days. Array CGH analysis was carried out for ME-CRCs and ME-non CRCs to determine if the CRC system impacted the genomic profile of the cells. This analysis indicated no major changes in the normal genome copy number composition of the CRCs at P5 (Figure S2A and B). At later passages, despite the fact the morphology of the cells appeared normal (Figure 1B), there was an increased level of copy number alterations, with gains and losses on chromosomes 6, 11, and 18 at P18, and chromosomes 1, 3, 4, 7, 9, 13, 14, and 18 at P76, respectively (Figure S2C and D). We next investigated whether the later passage mouse CRCs were able to maintain the differentiation properties of normal ME cells when grown in 3D Matrigel culture. In Matrigel, the ME-CRCs formed acinar structures with a well-defined cell/Matrigel interface and showed polarized synthesis of laminin around the periphery of the colony, similar to that observed with untransformed MCF10A human ME cells (Figure 1C). Despite the increase in aneuploidy, the number of spheres observed in Matrigel cultures in late vs. early passage ME-CRCs was unchanged (Figure 1D). Thus, with serial passage, the ME-CRCs maintain the ability to differentiate into a normal phenotype and regenerate mammary acinar structures similar to those formed by normal cells, in 3D Matrigel culture.

### Conditionally Reprogramming ME Cells Restores Expression of Progenitor-Associated Cell Surface Markers

**Figure 2:**
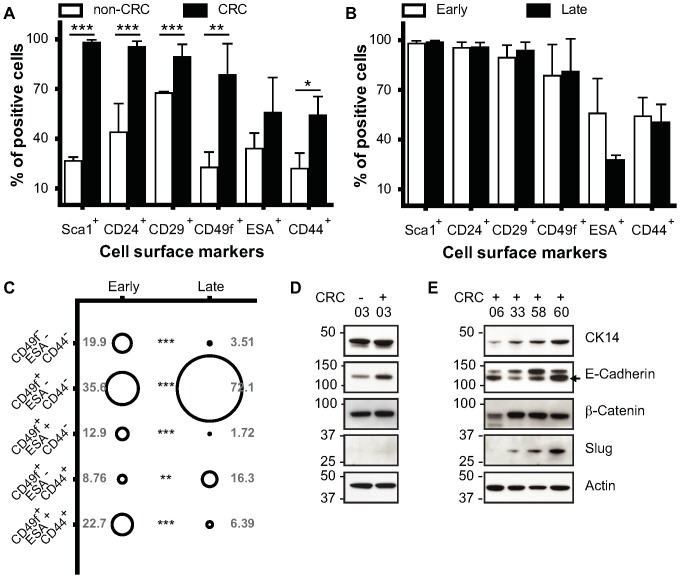
Expression of cell surface markers in ME-CRCs. Monoparametric FACS analysis of expression of indicated markers in freshly isolated ME non-CRCs and early passage ME-CRCs **A**) and early and late passage ME-CRCs **B**). **C**) Three-marker profile identified by multiparametric FACS analysis in early and late passage cells. **D and E**) Representative western blots showing expression of the indicated markers in non-CRCs (−) and CRCs (+) at the indicated passages. Values are mean±SEM of three independent experiments; ***P<0.001, **P<0.01, *P<0.05.

Human keratinocytes, when exposed to the CRC system, assume a stem-like state [5]. We investigated whether this was also true of mouse ME-CRCs. We assessed the individual and concomitant expression of the cell surface markers Sca1 (stem cell antigen), CD24 (luminal cell adhesion P-selectin), CD29 (β1 integrin), CD49f (α6 integrin), ESA (epithelial specific marker Epcam), and CD44 (hyaluronic acid receptor), which are differentially expressed in mouse mammary progenitor (CD49f, CD44, and Sca1) cells vs. differentiated luminal (CD24 and ESA) and myoepithelial (CD29 and CD49f) cells [17], [18]. To determine whether expression levels of these markers changed with conditional programming, we carried out FACS analysis of live cells (Figure S1B). The expression of each cell surface marker in freshly-isolated (non-CRC) ME cells was compared with that in P3–7 ME-CRCs (Figure 2A). As shown in Figure 2A, with exposure to the CRC system, there was an initial rapid rise in the number of cells individually expressing Sca1, CD24, CD29, or CD49f, with a less dramatic increase in cells expressing either ESA or CD44. For Sca1 and CD49f, there were also initial increases in overall protein expression per cell that were sustained in later passages (Figure S1D and E). Also at later passages (>P10) the expression of most cell surface markers remained elevated, with a large number of ME-CRCs expressing Sca1^+^, CD24^+^, CD29^+^, and CD49f^+^ (Figure 2B). Of note is that the major difference between early and late passage ME-CRCs is a greater than 50% decrease in the number of cells expressing ESA (Figure 2B), returning to levels seen in non-CRC ME cells. To analyze these subpopulations further, multiparametric FACS analysis was performed. The greatest change was in cells expressing ESA; e.g., the level of CD49f^+^/ESA^+^/CD44^+^ cells dropped from approximately 23% to 6% between early and late passage (Figure 2C). Consistent with this, we observed significant increases in the CD49f^+^/ESA^−^/CD44^+^ and CD44^+^/ESA^−^/CD49f^+^ subpopulations in late vs. early ME-CRCs (Figures 2C and S3H). We also noted that some ME-CRC cells that express Sca1, such as the Sca1^+^/CD24^+^/CD29^+^ or Sca1^+^/CD24^+^/CD49f^+^ subpopulations are equally expressed at a level greater than 80% at early and late passages (Figure S3A–F). The subsets of mouse mammary progenitor cells expressing CD29^+^/Sca1^−^/CD24^+^ or CD49f^+^/Sca1^−^/CD24^+^ were unchanged by serial passage (Figure S3C and F), but represented less than 1% of the population of ME-CRCs. These subsets are of interest because cells expressing similar patterns of markers can generate ductal outgrowths upon mammary fat pad transplantation [19], [20]. Overall, the results of FACS analysis suggest that serial passage does not affect some progenitor-like populations, while depleting others. In addition, a substantial number of differentiated cells are present in the ME-CRC population at early and late passage.

### ME-CRCs Do Not Undergo Epithelial to Mesenchymal Transition (EMT)

EMT induction with TGFβ or overexpression of EMT factors Snail or Twist can increase the number of cells with a stem-like profile [21], [22]. To investigate whether the CRC system was inducing changes in progenitor characteristics through induction of EMT, we investigated the intracellular expression of various markers of differentiation and EMT [23]. Protein expression patterns were examined in whole cell extracts of early- and late-passage CRCs and compared to the expression patterns in P3 cells that had not been exposed to the CRC system. Despite the fact that Slug expression increased at later passages (Figure 2D and E), we found that E-cadherin levels also increased between early (P6) and late (P33 and 58) passage (Figure 2D and E), suggesting that EMT is not occurring. β-catenin expression has also been associated with mammary stem cells [17]. However, β-catenin expression level was not changed on exposure to the CRC system or upon serial CRC passage (Figure 2D and E) and N-cadherin and vimentin levels were below detection limits in normal ME-CRCs (data not shown). Consistent with results in human CRCs [5] we found no evidence of reversion of ME-CRCs to pluripotent progenitors, as demonstrated by the fact that markers, such as Nanog and Oct 4, were not detected by western blotting at any passage (data not shown). Overall, the results of western analysis of ME-CRC protein expression are consistent with the preliminary conclusions drawn from the morphological and FACS data (Figures 1, 2 and S3), indicating that, as the ME cells are passaged in the CRC system, they maintain their epithelial phenotype. However, there is enrichment of cells that exhibit progenitor expression patterns (Figures 1, 2 and S3). This cell-type distribution does not change dramatically with serial CRC passage, despite increases in aneuploidy in the mouse ME-CRCs. There are also clear changes in gene expression in the later-passage ME-CRCs, most notably in the cell surface luminal differentiation markers Sca1 and ESA.

### Rho kinase inhibitor permanently reprogrammed mouse mammary cells

**Figure 3:**
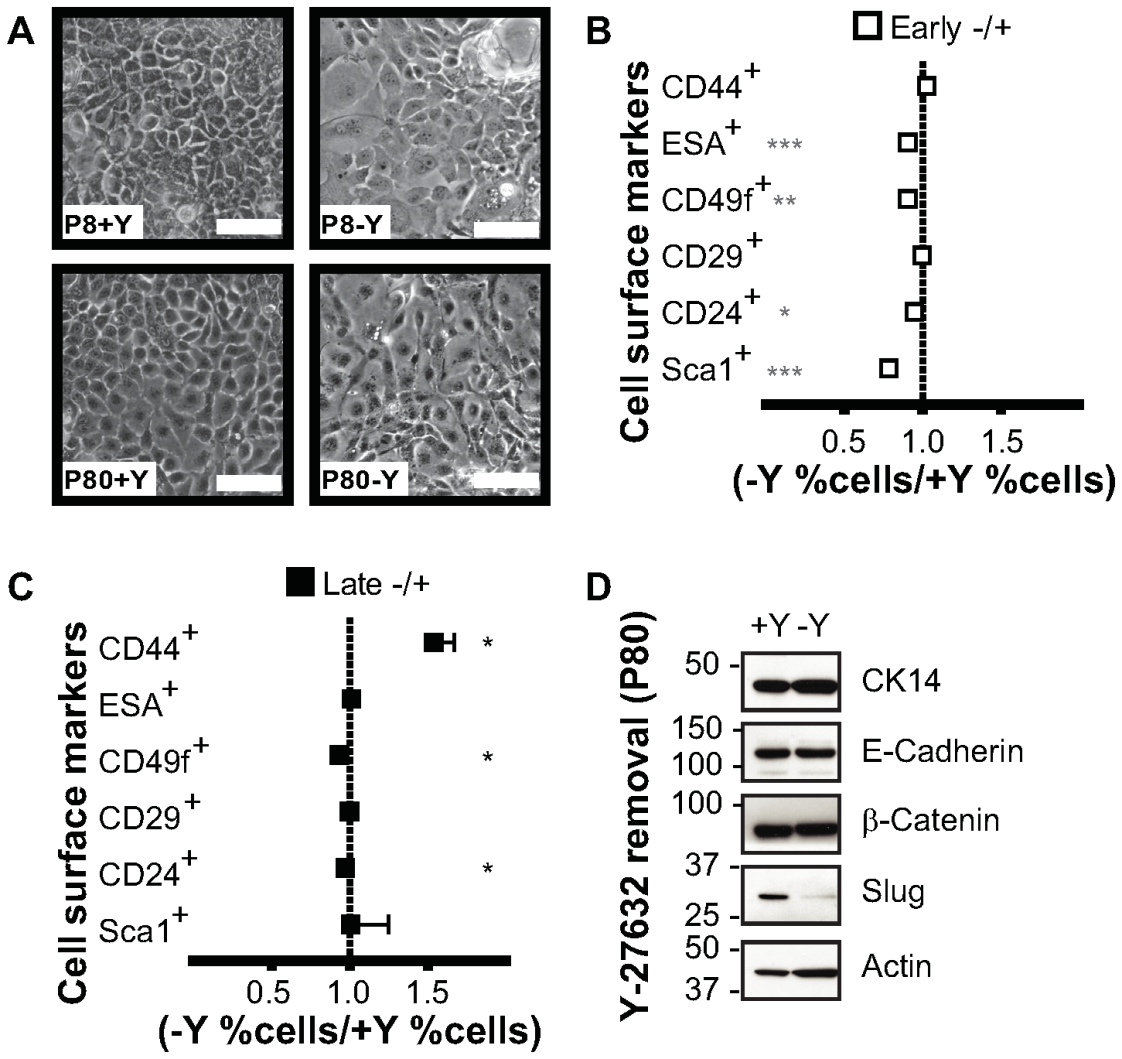
Removal of Y-27632 from the ME-CRCs alters expression of some cell surface markers. Early and late passage ME-CRCs were seeded on irradiated feeders with (+Y) or were cultured under CRC conditions (+Y), followed by the removal of Y-27632 and culture for two more passages (-Y). **A**) Phase contrast images of cells at indicated passages; magnification 10×; scale, 100 µm. **B and C**) Monoparametric FACS analysis of expression of the indicated markers in (**B**) early and (**C**) late passage cells. Values are mean±SEM of three independent experiments; ***P<0.001, **P<0.01, *P<0.05. **D**) Representative western blots of indicated markers expressed in P80 ME-CRCs under conditions of (+Y) and (-Y).

We next examined whether the effects of Rho kinase inhibitor Y-27632 on mouse ME cells were reversible by co-culturing early and late passage ME-CRCs with feeder cells in the absence of Y-27632 for two passages. Cells cultured in the absence of the inhibitor proliferated more slowly, but the overall cobblestone morphology was unaltered two weeks after withdrawal of Y-27632 (Figure 3A). Consistent with the minimal morphological change, expression levels of most ME-CRC cell surface markers in the absence of Y-27632 were unaltered or minimally changed at early or late passage (Figure 3B and C); however, CD44 levels were significantly increased in the absence of Y-27632 at late passage (Figure 3C). Conversely, Sca1 levels were reduced in the early passage cells after Y-27632 withdrawal (Figure 3B). Examination of cellular protein expression after Y-27632 withdrawal indicated that the cells continued to express E-cadherin, although Slug was decreased (Figure 3D). Our data indicate that many, but not all, cell surface hallmarks of a progenitor phenotype are maintained in the mouse ME cells with culture in the absence of Y-27632 for 2 weeks.

### Conditional Reprogramming of Mouse Mammary Tumor Epithelial Cells

**Figure 4:**
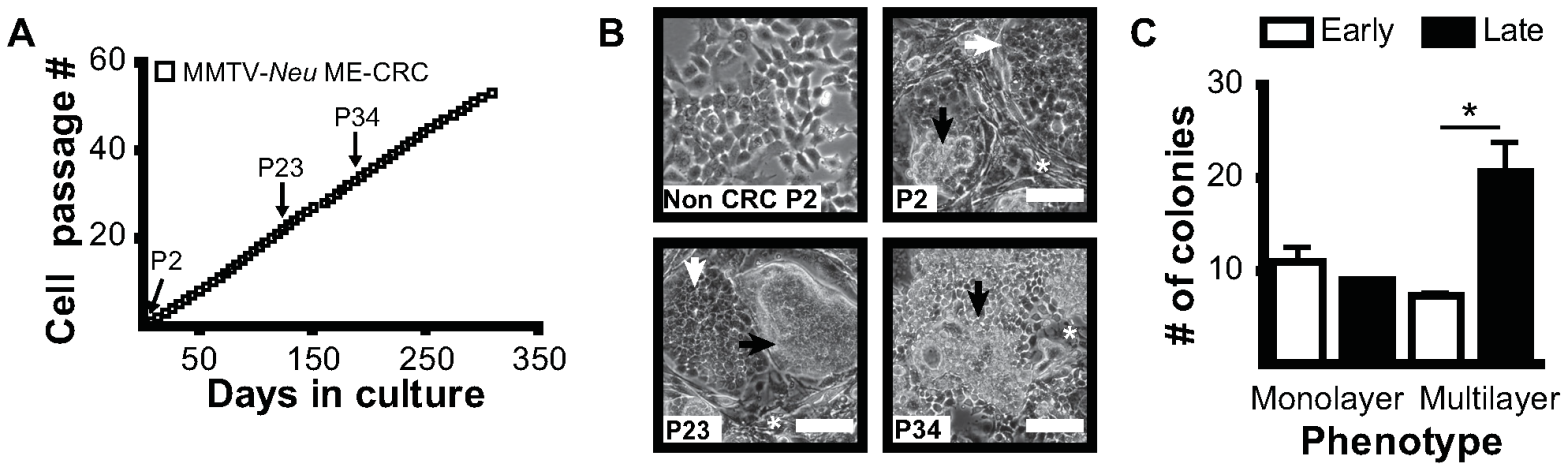
Properties of MMTV-*Neu* ME-CRCs in culture. **A**) Culture of tumor epithelial cells from a spontaneous MMTV-*Neu* mouse mammary gland tumor in the CRC system. **B**) Phase contrast images of MMTV-*Neu* ME-CRCs and non-CRCs at indicated passages on day 5 of culture (10×; scale, 100 µm). **C**) Quantification of colony subtypes of early (P2) vs. late (P23) passage MMTV-*Neu* ME-CRCs. Values are mean±SEM of three independent fields. *P<0.05.

**Figure 5:**
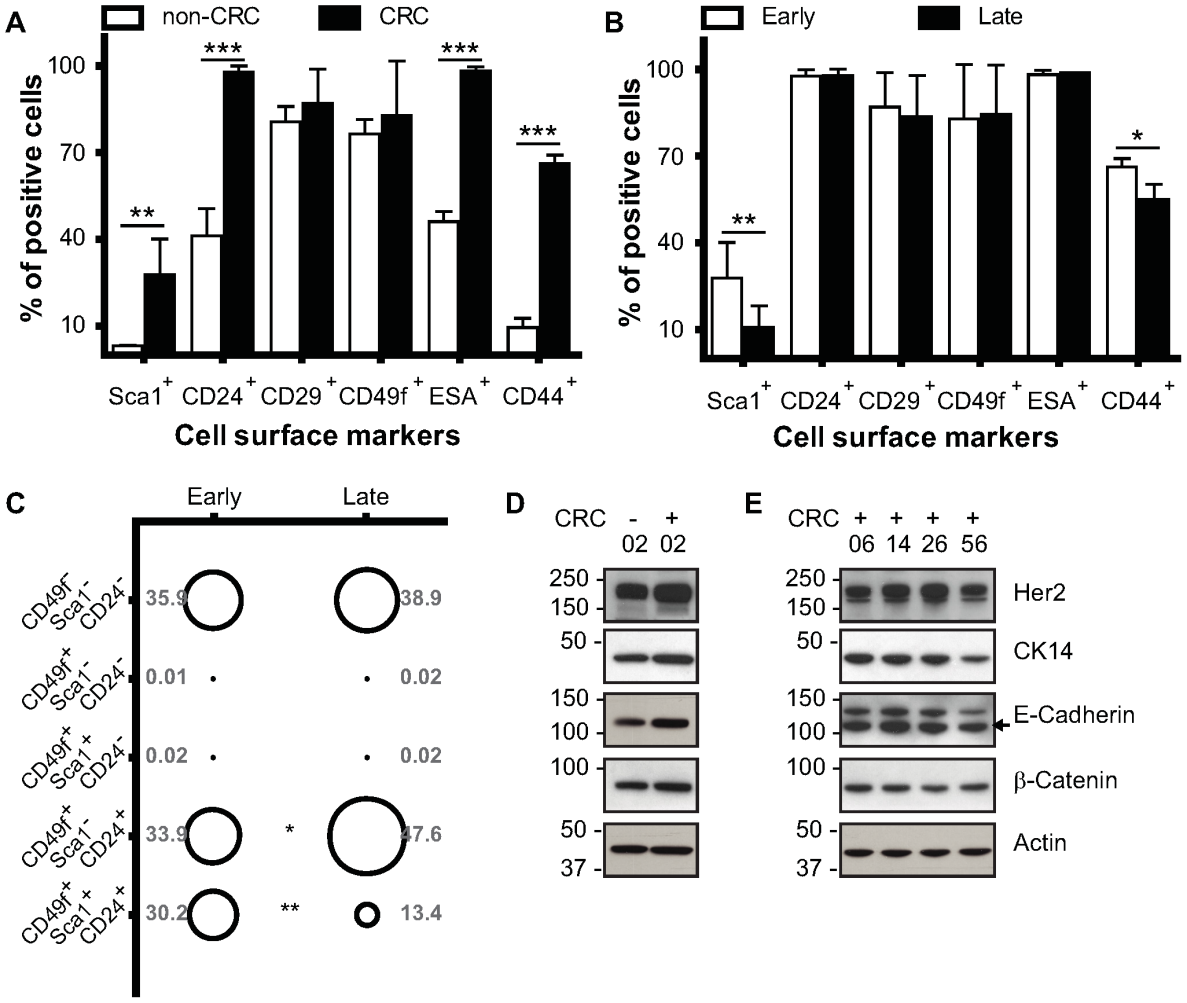
Cell surface marker expression in MMTV-*Neu* ME-CRCs. **A**) Monoparametric FACS analysis of expression of indicated markers in freshly isolated MMTV-*Neu* ME non-CRCs and early passage MMTV-*Neu* ME-CRCs and **B**) early and late passage MMTV-*Neu* ME-CRCs. **C**) Three-marker profile identified by multiparametric FACS analysis in early and late passage cells. **D**) & **E**) Representative western blots showing expression of the indicated markers in non-CRCs (−) and CRCs (+) at the indicated passages. Values are mean±SEM of three independent experiments; ***P<0.001, **P<0.01, *P<0.05.

To determine if transformed mouse ME-CRCs could be generated, we isolated epithelial cells from mammary tumors of MMTV-*Neu* mice [14]. In this model, wild-type *Neu* is overexpressed in the mammary glands, resulting in an activating transmembrane mutation in the *Neu* transgene that promotes mammary tumorigenesis [15]. This model closely mimics the progression of human breast cancer driven by the amplification and overexpression of the human homologue of HER2/*neu* (ErbB2). At an average age of 9 months, MMTV-*Neu* mice develop highly vascular mammary adenocarcinomas [14]. Short-term epithelial cultures can be prepared from cells isolated from these tumors but, under our culture conditions, a large portion of the cells senesce after several passages (Figure S4A). In contrast, ME-CRCs generated from MMTV-*Neu* tumors were serially passaged in the CRC system for >50 passages and did not senesce (Figure 4A). Unlike the relatively stable morphology of normal mouse ME-CRCs, the morphology of the MMTV-*Neu* ME-CRCs changed with serial passage, with an increase in multilayer colony-like structures at later passages (Figure 4B and C). We found also that there were chromosomal gains (chromosomes 2 and 12) and losses (chromosome 4) in these cells after extended passage (P38–73) but no changes are observed in early non-CRC (P2) and CRC (P6) passages, as determined by array CGH profiling (Figure S5A–D). To further investigate the characteristics of the MMTV-*Neu* MEC cells at early and late CRC passage, we determined the proportions of progenitor and differentiated cells by FACS analysis of cell surface markers known to be expressed by tumor-initiating cells in mouse models of mammary cancer [24] or by more differentiated tumor cells [17], [25]. We used monoparametric and multiparametric FACS approaches that were used for analysis of normal ME-CRCs (Figure S4B and C). An initial increase in the number of cells expressing Sca1, CD24, ESA, and CD44 (Figure 5A), as well as increased expression of each of these proteins per cell (Figure S4D and E) compared to non-CRC MMTV-*Neu* MECs freshly isolated from tumors was observed. Elevated levels of cells expressing these surface markers were sustained in late-passage MMTV-*Neu* ME-CRCs, with the notable exception of Sca1, which decreased by greater than 50% (Fig. 5B). Multiparametric FACS analysis indicated that early passage MMTV-*Neu* ME-CRCs showed no change in the ESA/CD44/CD49f subpopulation (Figure S6A–C). However, a significant change was the decrease in the CD49f^+^/Sca1^+^/CD24^+^ subpopulation which constituted <17% of the late passage MMTV-*Neu* ME-CRCs (Figures 5C and S6G–H). This combination of cell surface markers has been described as defining a HER2^+^ tumor-initiating population in some studies [26]–[29]. Of note, also, is that a large portion of the Sca1^+^ MMTV-*Neu* CRCs were also positive for CD24 and CD29 (Figure S6D–F) and, despite the overall decrease in Sca1^+^ cells, the CD24^+^/CD29^+^/Sca1^+^ subpopulation still constitutes only approximately 9% of the cell population at later passages (Figure S6D–F). Western analysis of lysates of late-passage MMTV-*Neu* ME-CRCs showed that HER2/*neu* continued to be expressed (Figure 5D and E). Interestingly, despite the morphological changes in the MMTV-*Neu* ME-CRCs, there were no major changes in expression of EMT markers, such as E-cadherin and β-catenin, which continued to be expressed at similar levels at early and late passage. Similar to the normal ME-CRCs, there was no detectable expression of pluripotent progenitor markers at any of the passages examined (data not shown).

### Reprogramming of MMTV-*Neu* ME-crcs is Partially Reversed by Removal of Y-27632

**Figure 6:**
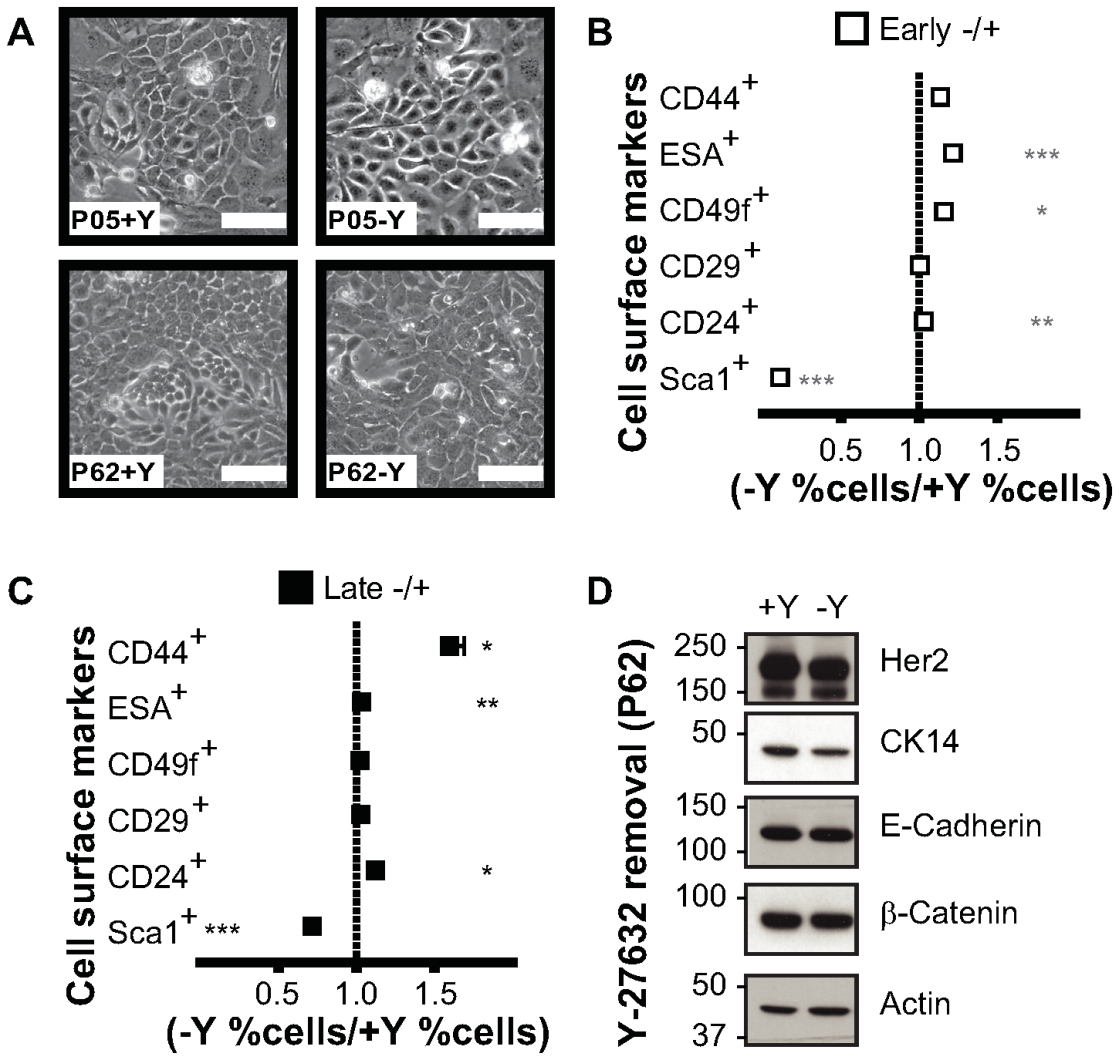
Removal of Y-27632 from MMTV-*Neu* ME-CRCs alters expression of some cell surface markers. Early and late passage MMTV-*Neu* ME-CRCs were seeded on irradiated feeders with (+Y) or were cultured under CRC conditions (+Y), followed by the removal of Y-27632 and culture for two more passages (-Y). **A**) Phase contrast images of cells at indicated passages. **A**) Phase contrast images (10×; scale, 100 µm). **B and C**) Monoparametric FACS analysis of expression of the indicated markers in early (**B**) and late (**C**) passage cells. Values are mean±SEM of three independent experiments; ***P<0.001, **P<0.01, *P<0.05. **D**) Representative western blots of indicated markers expressed in P62 ME-CRCs under conditions of (+Y) and (-Y).

We also determined if removal of the inhibitor Y-27632 from the MMTV-*Neu* ME-CRC cultures caused changes in the populations of cells at early or late passages. The proliferation of early-passage ME-CRCs was reduced by Y-27632 withdrawal, but the overall morphology of the cells did not change significantly (Figure 6A). FACS analysis of cell surface markers revealed that Y-27632 withdrawal from early- and late-passage MMTV-*Neu* ME-CRCs reduced the number of cells expressing Sca1, while significantly increasing the number of ESA^+^ and CD49f^+^ cells in early passage and the CD44^+^ and CD24^+^ cells in late passage (Figure 6B and C). Western analysis of cell lysates revealed that MMTV-*Neu* CRCs cultured in the presence or absence of Y-27632 maintained expression of HER2/*neu*, E-cadherin, and β-catenin (Figure 6D). Overall, these data suggest that there is some permanent reprogramming of the MMTV-*Neu* ME-CRCs with continued passage in the CRC system, although other changes in gene expression that are dependent on Y-27632 are reversible.

### MMTV-*Neu* ME-crcs form Mammary Tumors *in vivo*

**Figure 7:**
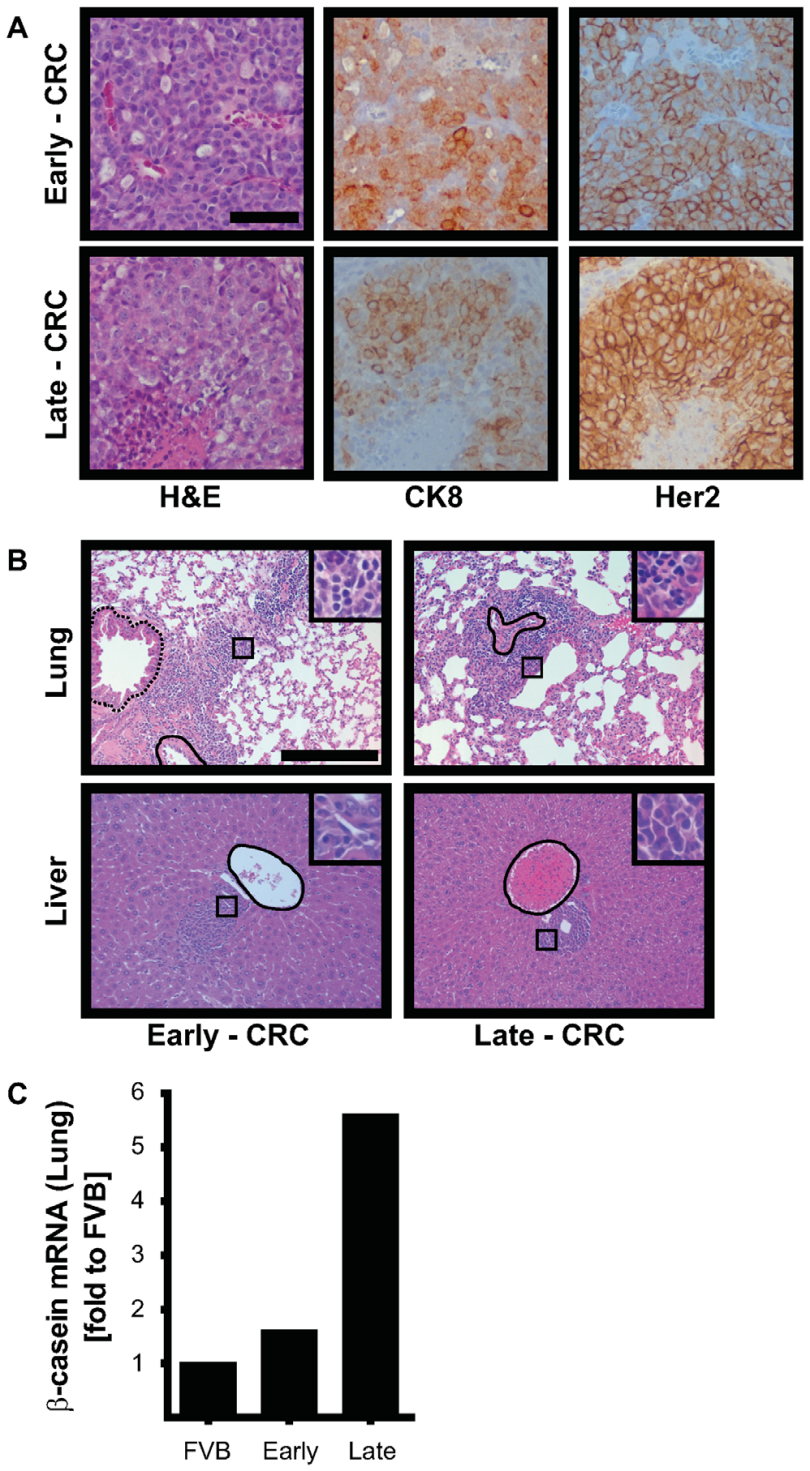
Mammary tumor formation after orthotopic transplantation of MMTV-*Neu* ME-CRCs into syngeneic mice. **A**) H&E and Immunohistochemical staining of CK8 and Her2 in early (P6) and late (P68) MMTV-*Neu* ME-CRC-derived tumors. (20×; scale, 50 µm). **B**) Metastasis-like lesions in H&E-stained lung and liver of mice with primary mammary tumors derived from early and late CRCs. Blood vessels and bronchi are outlined. (20×; scale, 200 µm). **C**) qRT PCR analysis of β-casein mRNA assessed in extracts of normal (FVB) lung or lungs of mice harboring tumors derived from early or late passage MMTV-*Neu* ME-CRCs.

To determine their tumorigenic potential, we injected early- and late-passage MMTV-*Neu* ME-CRCs into the mammary fat pads of syngeneic mice. After approximately 6 weeks, large tumors had developed and the mice were sacrificed. The histopathology of the early and late passage MMTV-*Neu* ME-CRC allograft tumors was indistinguishable from previous descriptions of tumors that occur in the MMTV-*Neu* parental mice at approximately 7–9 months of age [14] (Figure 7A). These are highly vascular, largely cribriform adenocarcinomas, with necrotic areas and increased mitotic figures that stain uniformly for high expression of the oncogene Her2/*neu and* CK8 (Figure 7A). The transgenic MMTV-*Neu* mice also developed metastases at a later stage to the lung and liver [15]. We found that a similar metastatic pattern, with extensive lymphocyte infiltration, occurred in both lung and liver of the mice harboring the transplanted MMTV-*Neu* ME-CRCs (Figure 7B). The presence of ME cells in these metastatic lesions was confirmed by detection of mammary-specific β-casein mRNA by qRT-PCR (Figure 7C). Similar results were obtained with MMTV-*Neu* ME-CRCs implanted in nude mice (data not shown).

## Discussion

Here, we show that the CRC system [3] can be used for indefinite passage of normal and transformed mouse ME cells. There are similarities and notable differences between human and mouse CRCs. Both human and mouse epithelial CRCs seem to assume a progenitor-like phenotype, at least as assessed by changes in cell surface markers, such as the integrin family members CD29 and CD49f, and the cell adhesion molecules CD24 and CD44. Neither the human nor the mouse CRCs revert to pluripotent progenitor cells, nor do they undergo mesenchymal transition. Normal human conditionally reprogrammed keratinocytes maintain tissue-specific differentiation potential and have been shown to form a stratified epithelial layer in a 3D culture system [5]. Likewise, the normal ME-CRCs can assume a mammary acinar-like phenotype when grown in a 3D matrix of Matrigel. MMTV-*Neu* ME-CRCs are able to form tumors when transplanted into mice, and these tumors are histopathologically indistinguishable from the tumors, and the metastases, that evolve in the MMTV-*Neu* transgenic animals. Thus, there is significant overlap in the properties of mouse and human CRCs.

A notable difference between the mouse and human CRCs is the response to withdrawal of the Rho kinase inhibitor Y-27632. It has been shown that removal of this compound from human keratinocyte CRC cultures causes complete reversion to the non-CRC phenotype [5]. In contrast, withdrawal of Y-27632 did not alter the expression of many of the genes associated with mouse mammary progenitor cells. We conjecture that the reason for this may lie in fundamental differences in the regulation of the reprogramming phenomenon in mouse and human CRCs *in vitro*. Despite this, the lack of phenotypic reversion in the mouse CRCs does not appear to impede the cells from driving the correct differentiated phenotype *in vivo*. This suggests that the ME-CRCs, when exposed to components of the normal stromal environment, are capable of undergoing differentiation.

Differences in stem-like markers were observed, however, with increasing passage number in both normal and transformed ME-CRCs. In the normal ME- CRCs, frequency of expression of the cell surface marker ESA reverts to that of non-CRC cells as the cells are serially passaged; in particular, ESA^+^, CD44^+^ CD49f^+^ subpopulation is significantly decreased with progressive passage. Although the reduction in ESA^+^ cells does not impede formation of mammary acinar structures in Matrigel, it might impede other aspects of normal mammary outgrowth development *in vivo*. With serial passage of MMTV-*Neu* ME-CRCs, we observed loss of Sca1 expression by the cells. Loss of Sca1^+^ cells does not appear to impede tumor formation *in vivo*, because tumors formed efficiently with both late and early passage MMTV-*Neu* ME-CRCs. Sca1 has been described as an important marker of tumor-initiating cells in luminal breast cancer [30]. It may be that the HER2/*neu*-driven subtype of breast cancer is less dependent on Sca1^+^ tumor-initiating cells than other breast cancer subtypes. Overall, the properties of early and late passage MMTV-*Neu* ME-CRCs indicate that a population of tumor-initiating cells is maintained. Despite the gross phenotypic and morphological differences in the cells grown in the CRC system, MMTV-*Neu* ME-CRCs retain a subpopulation of tumor-initiating cells that are capable of tumor formation and metastasis indistinguishable from that seen in the parental MMTV-*Neu* mice.

With increasing passage, an increase in aneuploidy in both normal ME-CRCs and MMTV-*Neu* CRCs was observed. The phenomenon whereby tumor cells show increased chromosomal rearrangement and aneuploidy with passage *in vitro* has been described for many cancer cell lines. It is not possible to compare the aneuploidy of the same late-passage normal and transformed ME-CRCs with the respective non-CRC cultures, because the latter senesce. However, the aneuploidy observed could be the result of *in vitro* changes that occur with increasing passage of most cells in culture, and may not be specific to the CRC system. That said, clearly, the early and late passage CRCs are not populations of genetically identical cells and, for any phenotype or response obtained, it will be important to define the cell passage number at which it is observed.

A major question arising from this work is how both normal and transformed ME-CRCs bypass senescence check points. Previous studies have utilized Rho kinase inhibitors for immortalization of stem cells and suggested that this function is mediated, in part, through effects on integrin and adhesion signaling [31]. Rho kinase-dependent EMT has also been described previously in mouse mammary cancer cells [32]. However, mesenchymal differentiation does not occur in the normal mouse CRCs. Of note is that the conditional reprogramming does not occur in the ME cells unless both the irradiated fibroblasts and the Y compound are present (data not shown) and [5]. This suggests that the irradiated fibroblasts in the CRC system likely provide a secreted or adhesion factor that initiates or modifies the Rho kinase effect. Consistent with this, recent data indicates that conditioned medium from the irradiated fibroblasts can be used in the CRC system instead of the irradiated cells [33].

## Conclusion

Overall, the ability to conditionally reprogram and indefinitely propagate human and mouse epithelial cells could be widely used in experimental systems. The ability to carry CRCs from valuable transgenic sources and use them as transplants in syngeneic animals increases experimental efficiency and allows interactions with the stromal environment to be examined in detail. The utility of normal mouse epithelial cells for development studies also holds great promise.

## Supporting Information

Figure S1 **Expansion and flow cytometric analysis of normal mouse ME-CRCs.**
(TIFF)

Figure S2 **Comparative genomic hybridization of ME-CRCs.**
(TIFF)

Figure S3 **Multiparametric FACS analysis of cell surface markers expression in normal mouse ME-CRCs.**
(TIFF)

Figure S4 **Expansion and FACS analysis of MMTV-Neu ME-CRCs.**
(TIFF)

Figure S5 **Comparative genomic hybridization of MMTV-Neu ME-CRCs.**
(TIFF)

Figure S6 **Multiparametric FACS analysis of cell surface markers expression in MMTV-Neu ME-CRCs.**
(TIFF)

## References

[1] Kuilman T, Michaloglou C, Mooi WJ, Peeper DS (2010) The essence of senescence. Genes Dev 24: 2463–2479. 21078816 10.1101/gad.1971610 PMC2975923

[2] Roig AI, Eskiocak U, Hight SK, Kim SB, Delgado O, et al (2010) Immortalized epithelial cells derived from human colon biopsies express stem cell markers and differentiate in vitro. Gastroenterology 138: 1012–1021.e1011–1015. 19962984 10.1053/j.gastro.2009.11.052

[3] Liu X, Ory V, Chapman S, Yuan H, Albanese C, et al (2011) ROCK inhibitor and feeder cells induce the conditional reprogramming of epithelial cells. Am J Pathol 180: 599–607. 22189618 10.1016/j.ajpath.2011.10.036 PMC3349876

[4] Yuan H, Myers S, Wang J, Zhou D, Woo JA, et al (2012) Use of reprogrammed cells to identify therapy for respiratory papillomatosis. N Engl J Med 367: 1220–1227. 23013073 10.1056/NEJMoa1203055 PMC4030597

[5] Suprynowicz FA, Upadhyay G, Krawczyk E, Kramer SC, Hebert JD, et al (2012) Conditionally reprogrammed cells represent a stem-like state of adult epithelial cells. Proceedings of the National Academy of Sciences of the United States of America 109: 20035–20040. 23169653 10.1073/pnas.1213241109 PMC3523865

[6] Itahana K, Campisi J, Dimri GP (2004) Mechanisms of cellular senescence in human and mouse cells. Biogerontology 5: 1–10. 15138376 10.1023/b:bgen.0000017682.96395.10

[7] Sherr CJ, DePinho RA (2000) Cellular senescence: mitotic clock or culture shock? Cell 102: 407–410. 10966103 10.1016/s0092-8674(00)00046-5

[8] Wright WE, Shay JW (2000) Telomere dynamics in cancer progression and prevention: fundamental differences in human and mouse telomere biology. Nat Med 6: 849–851. 10932210 10.1038/78592

[9] Rosenfield SM, Bowden ET, Cohen-Missner S, Gibby KA, Ory V, et al (2012) Pleiotrophin (PTN) expression and function and in the mouse mammary gland and mammary epithelial cells. PLoS One 7: e47876. 23077670 10.1371/journal.pone.0047876 PMC3471873

[10] Debnath J, Brugge JS (2005) Modelling glandular epithelial cancers in three-dimensional cultures. Nat Rev Cancer 5: 675–688. 16148884 10.1038/nrc1695

[11] Lee GY, Kenny PA, Lee EH, Bissell MJ (2007) Three-dimensional culture models of normal and malignant breast epithelial cells. Nat Methods 4: 359–365. 17396127 10.1038/nmeth1015 PMC2933182

[12] Ory V, Tassi E, Cavalli LR, Sharif GM, Saenz F, et al. (2013) The nuclear coactivator amplified in breast cancer 1 maintains tumor-initiating cells during development of ductal carcinoma in situ. Oncogene. 10.1038/onc.2013.263 PMC3943533 23851504

[13] Oh A, List H-J, Reiter R, Mani A, Zhang Y, et al (2004) The nuclear receptor coactivator AIB1 mediates insulin-like growth factor I-induced phenotypic changes in human breast cancer cells. Cancer Res 64: 8299–8308. 15548698 10.1158/0008-5472.CAN-04-0354

[14] Fereshteh MP, Tilli MT, Kim SE, Xu J, O'Malley BW, et al (2008) The nuclear receptor coactivator amplified in breast cancer-1 is required for Neu (ErbB2/HER2) activation, signaling, and mammary tumorigenesis in mice. Cancer Res 68: 3697–3706. 18483252 10.1158/0008-5472.CAN-07-6702 PMC3641830

[15] Guy CT, Webster MA, Schaller M, Parsons TJ, Cardiff RD, et al (1992) Expression of the neu protooncogene in the mammary epithelium of transgenic mice induces metastatic disease. Proc Natl Acad Sci U S A 89: 10578–10582. 1359541 10.1073/pnas.89.22.10578 PMC50384

[16] Chapman S, Liu X, Meyers C, Schlegel R, McBride AA (2010) Human keratinocytes are efficiently immortalized by a Rho kinase inhibitor. J Clin Invest 120: 2619–2626. 20516646 10.1172/JCI42297 PMC2898606

[17] Li Y, Rosen JM (2005) Stem/progenitor cells in mouse mammary gland development and breast cancer. J Mammary Gland Biol Neoplasia 10: 17–24. 15886883 10.1007/s10911-005-2537-2

[18] Visvader JE (2009) Keeping abreast of the mammary epithelial hierarchy and breast tumorigenesis. Genes Dev 23: 2563–2577. 19933147 10.1101/gad.1849509 PMC2779757

[19] Stingl J, Eirew P, Ricketson I, Shackleton M, Vaillant F, et al. (2006) Purification and unique properties of mammary epithelial stem cells. Nature: 5. 10.1038/nature04496 16395311

[20] Shackleton M, Vaillant F, Simpson KJ, Stingl J, Smyth GK, et al (2006) Generation of a functional mammary gland from a single stem cell. Nature 439: 84–88. 16397499 10.1038/nature04372

[21] Mani SA, Guo W, Liao M-J, Eaton EN, Ayyanan A, et al (2008) The epithelial-mesenchymal transition generates cells with properties of stem cells. Cell 133: 704–715. 18485877 10.1016/j.cell.2008.03.027 PMC2728032

[22] Morel A-P, Lièvre M, Thomas C, Hinkal G, Ansieau S, et al (2008) Generation of breast cancer stem cells through epithelial-mesenchymal transition. PLoS One 3: e2888. 18682804 10.1371/journal.pone.0002888 PMC2492808

[23] Lee JM, Dedhar S, Kalluri R, Thompson EW (2006) The epithelial-mesenchymal transition: new insights in signaling, development, and disease. The Journal of Cell Biology 172: 973–981. 16567498 10.1083/jcb.200601018 PMC2063755

[24] Owens TW, Naylor MJ (2013) Breast cancer stem cells. Front Physiol 4: 225. 23986719 10.3389/fphys.2013.00225 PMC3753536

[25] Lindeman GJ, Visvader JE (2010) Insights into the cell of origin in breast cancer and breast cancer stem cells. Asia Pac J Clin Oncol 6: 89–97. 20565420 10.1111/j.1743-7563.2010.01279.x

[26] Matulka LA, Triplett AA, Wagner K-U (2007) Parity-induced mammary epithelial cells are multipotent and express cell surface markers associated with stem cells. Developmental Biology 303: 29–44. 17222404 10.1016/j.ydbio.2006.12.017

[27] Lo P-K, Kanojia D, Liu X, Singh UP, Berger FG, et al (2012) CD49f and CD61 identify Her2/neu-induced mammary tumor-initiating cells that are potentially derived from luminal progenitors and maintained by the integrin-TGFβ signaling. Oncogene 31: 2614–2626. 21996747 10.1038/onc.2011.439 PMC3260386

[28] Jeselsohn R, Brown NE, Arendt L, Klebba I, Hu MG, et al (2010) Cyclin D1 kinase activity is required for the self-renewal of mammary stem and progenitor cells that are targets of MMTV-ErbB2 tumorigenesis. Cancer Cell 17: 65–76. 20129248 10.1016/j.ccr.2009.11.024 PMC2818730

[29] Liu JC, Deng T, Lehal RS, Kim J, Zacksenhaus E (2007) Identification of tumorsphere- and tumor-initiating cells in HER2/Neu-induced mammary tumors. Cancer Res 67: 8671–8681. 17875707 10.1158/0008-5472.CAN-07-1486

[30] Batts TD, Machado HL, Zhang Y, Creighton CJ, Li Y, et al (2011) Stem cell antigen-1 (sca-1) regulates mammary tumor development and cell migration. PLoS One 6: e27841. 22140470 10.1371/journal.pone.0027841 PMC3226565

[31] Pakzad M, Totonchi M, Taei A, Seifinejad A, Hassani SN, et al (2010) Presence of a ROCK inhibitor in extracellular matrix supports more undifferentiated growth of feeder-free human embryonic and induced pluripotent stem cells upon passaging. Stem Cell Rev 6: 96–107. 20012714 10.1007/s12015-009-9103-z

[32] Castro DJ, Maurer J, Hebbard L, Oshima RG (2013) ROCK1 inhibition promotes the self-renewal of a novel mouse mammary cancer stem cell. Stem Cells 31: 12–22. 22961723 10.1002/stem.1224 PMC3967738

[33] Palechor-Ceron N, Suprynowicz FA, Upadhyay G, Dakic A, Minas T, et al (2013) Radiation induces diffusible feeder cell factor(s) that cooperate with ROCK inhibitor to conditionally reprogram and immortalize epithelial cells. Am J Pathol 183: 1862–1870. 24096078 10.1016/j.ajpath.2013.08.009 PMC5745544

