# Supplementary figures and images for: Conditionally Reprogrammed Normal and Transformed Mouse Mammary Epithelial Cells Display a Progenitor-Cell–Like Phenotype
Source: PLoS One. 2014 May 15;9(5):e97666. doi: 10.1371/journal.pone.0097666 (PMC4022745; doi:10.1371/journal.pone.0097666)

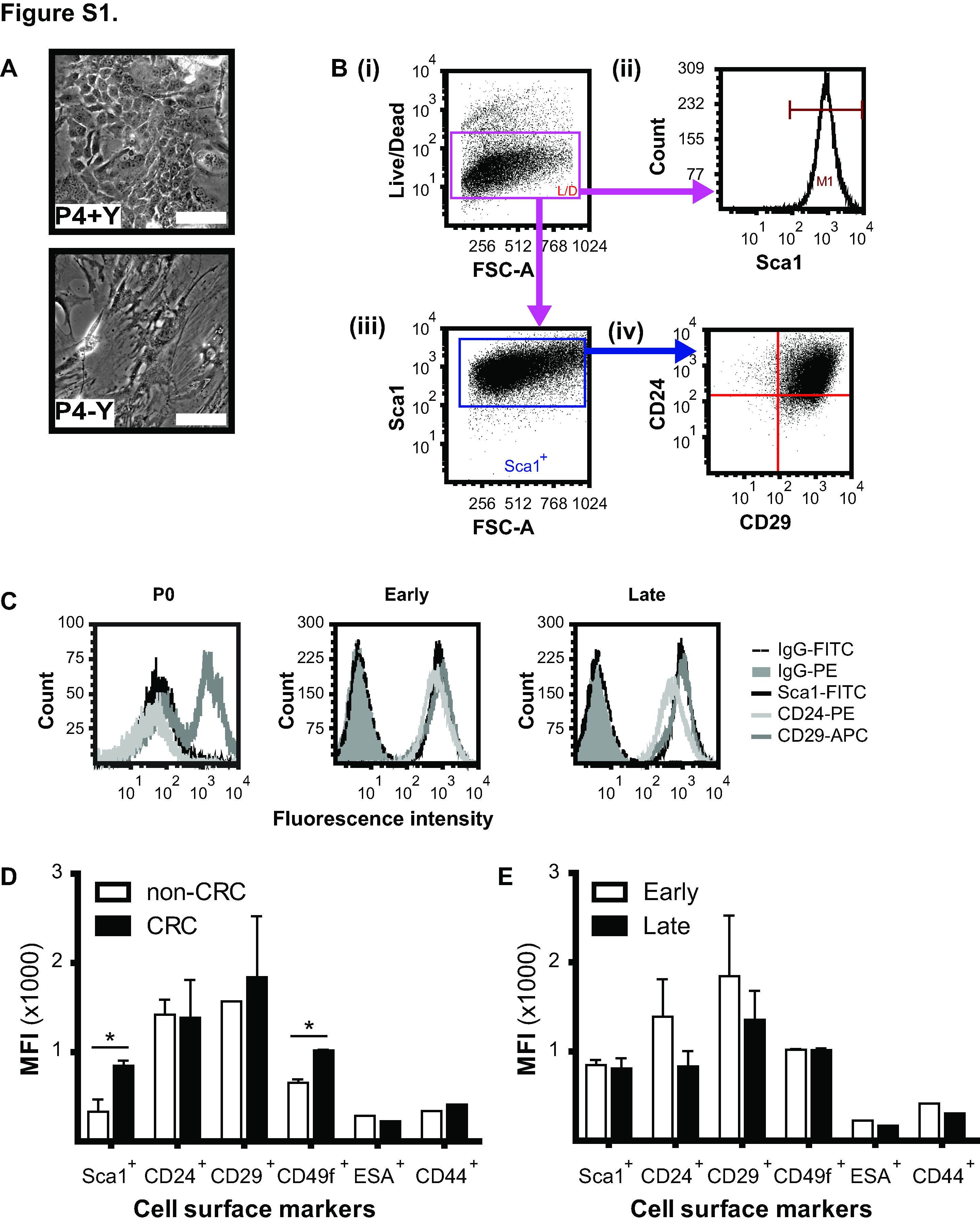

Supplement: Figure S1 — Expansion and flow cytometric analysis of normal mouse ME-CRCs. (TIFF) [file pone.0097666.s001.tif]

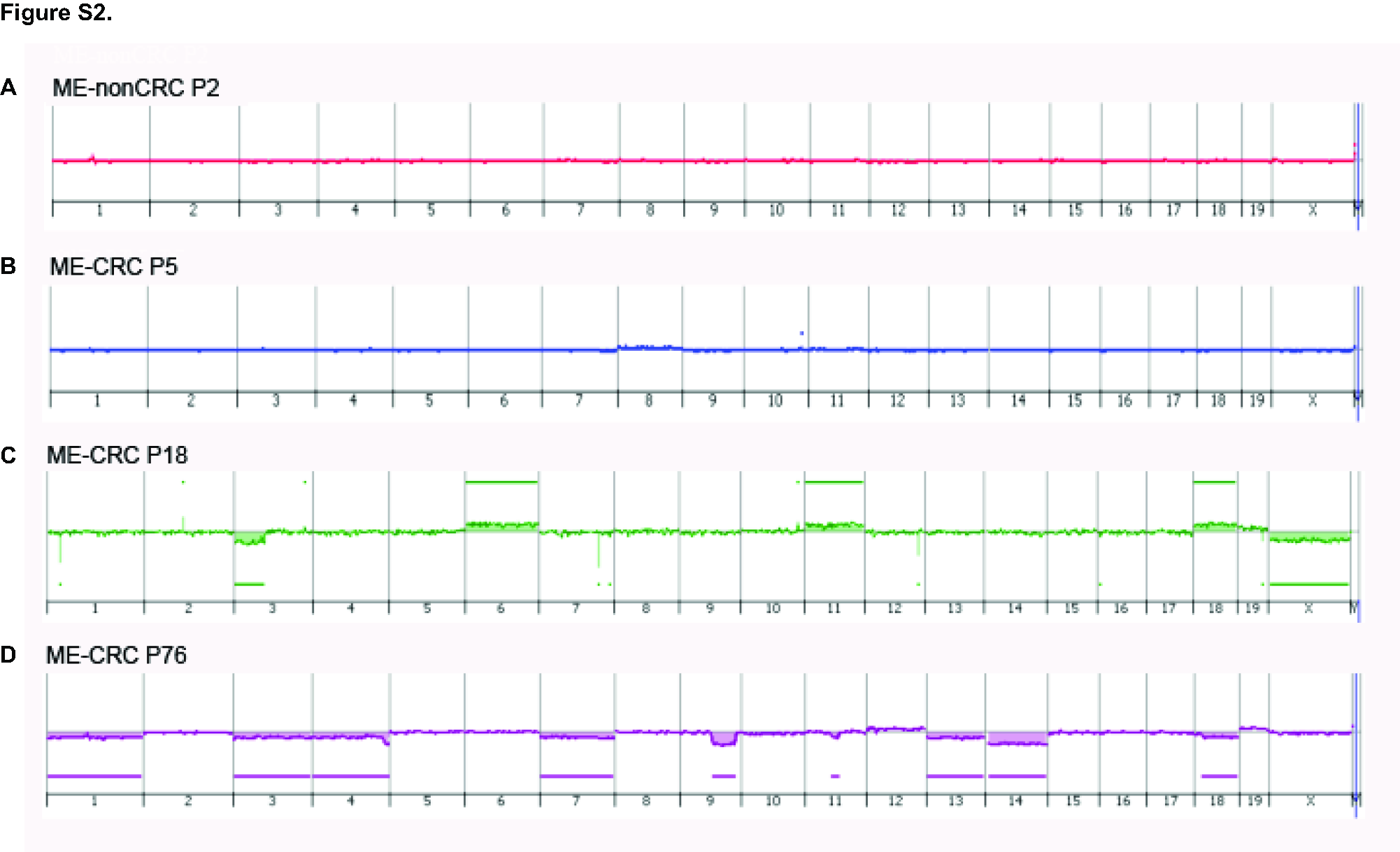

Supplement: Figure S2 — Comparative genomic hybridization of ME-CRCs. (TIFF) [file pone.0097666.s002.tif]

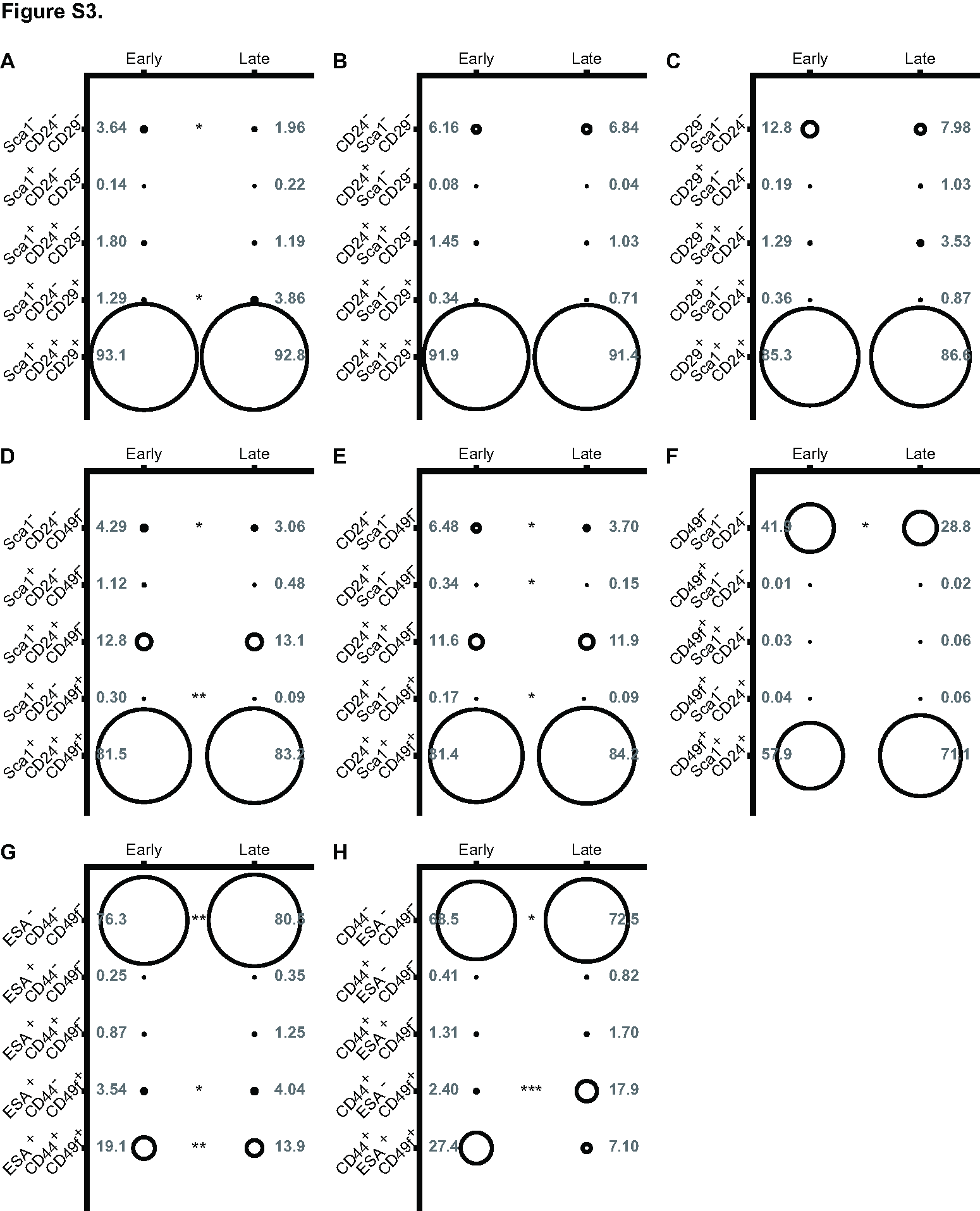

Supplement: Figure S3 — Multiparametric FACS analysis of cell surface markers expression in normal mouse ME-CRCs. (TIFF) [file pone.0097666.s003.tif]

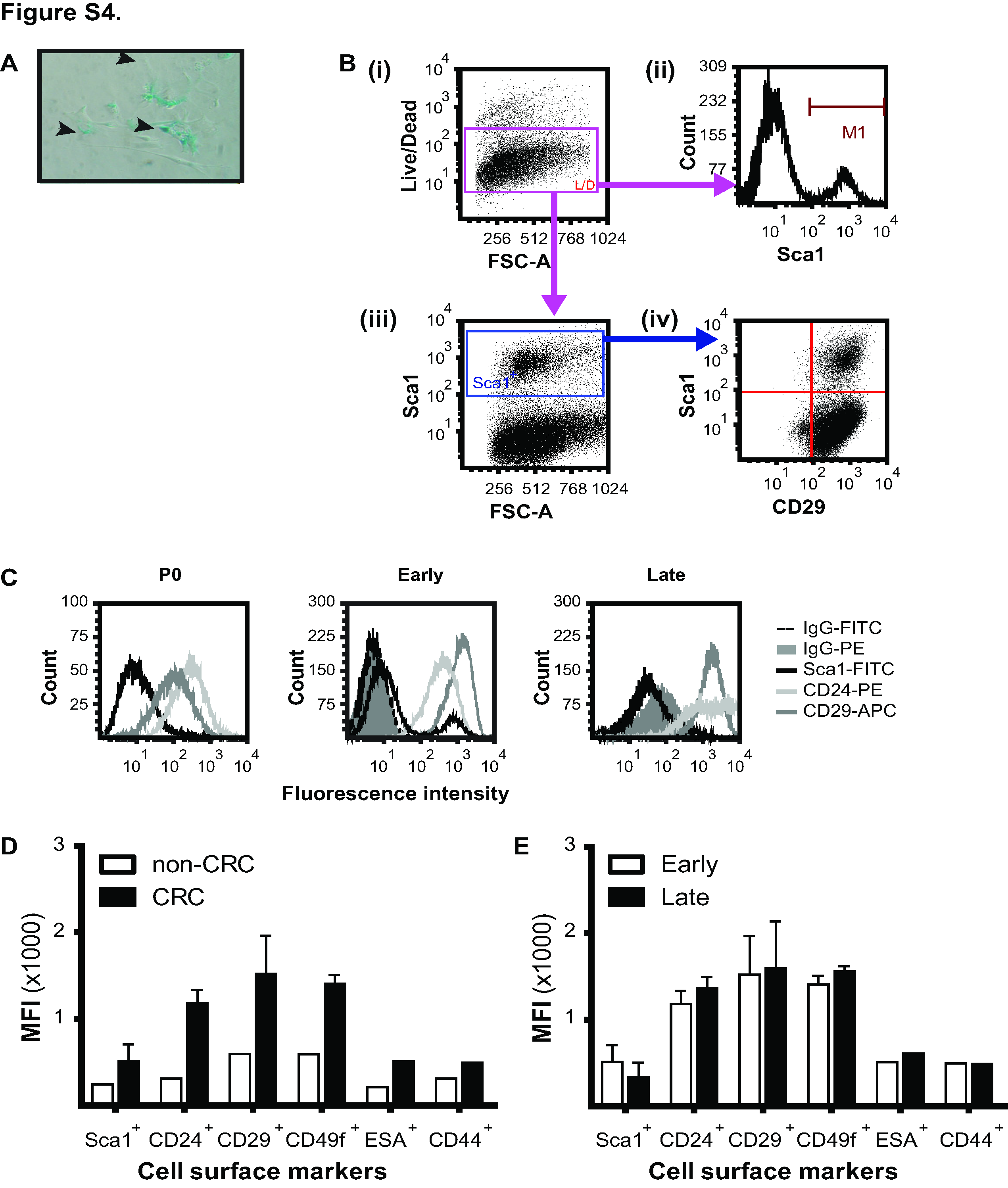

Supplement: Figure S4 — Expansion and FACS analysis of MMTV-Neu ME-CRCs. (TIFF) [file pone.0097666.s004.tif]

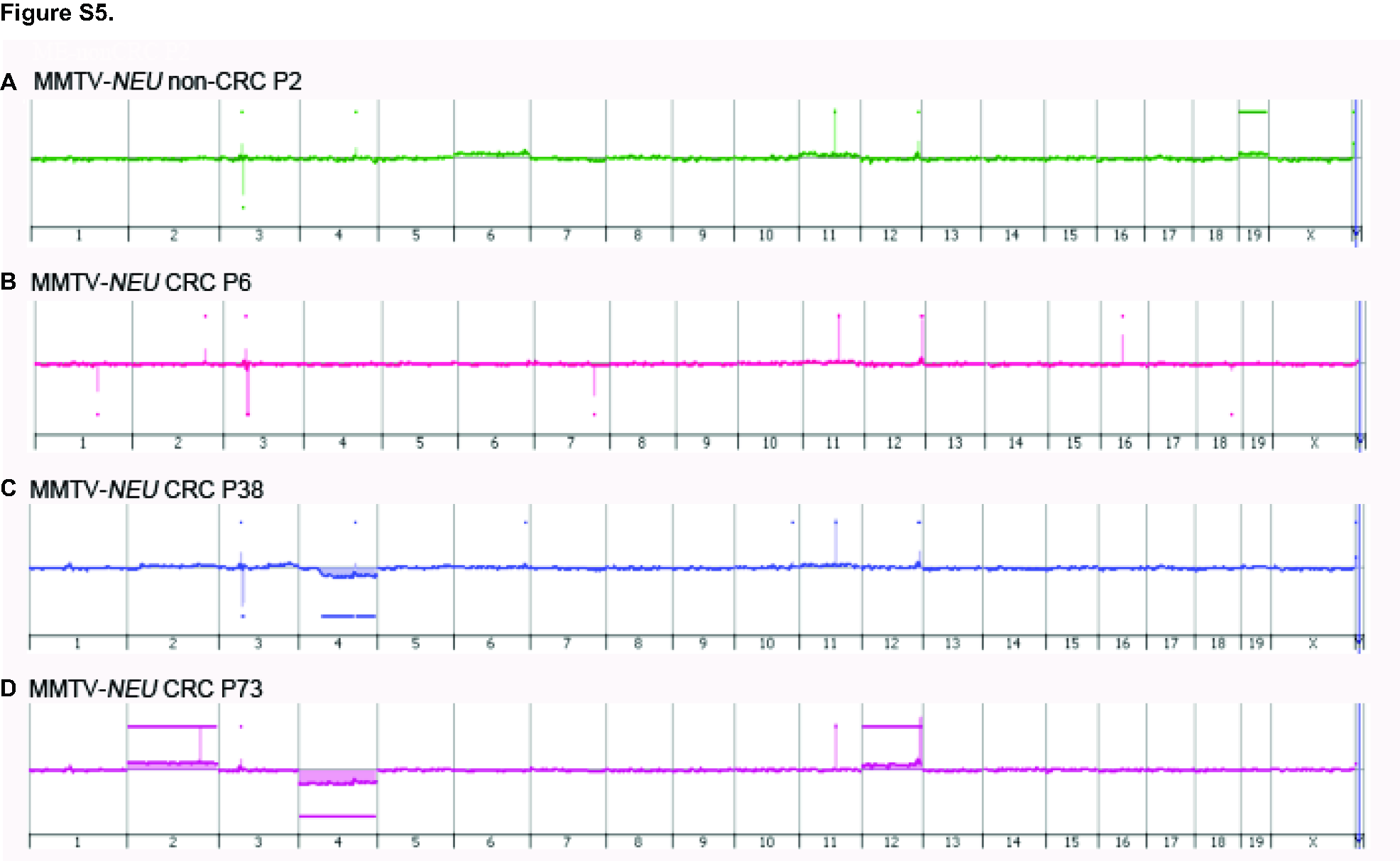

Supplement: Figure S5 — Comparative genomic hybridization of MMTV-Neu ME-CRCs. (TIFF) [file pone.0097666.s005.tif]

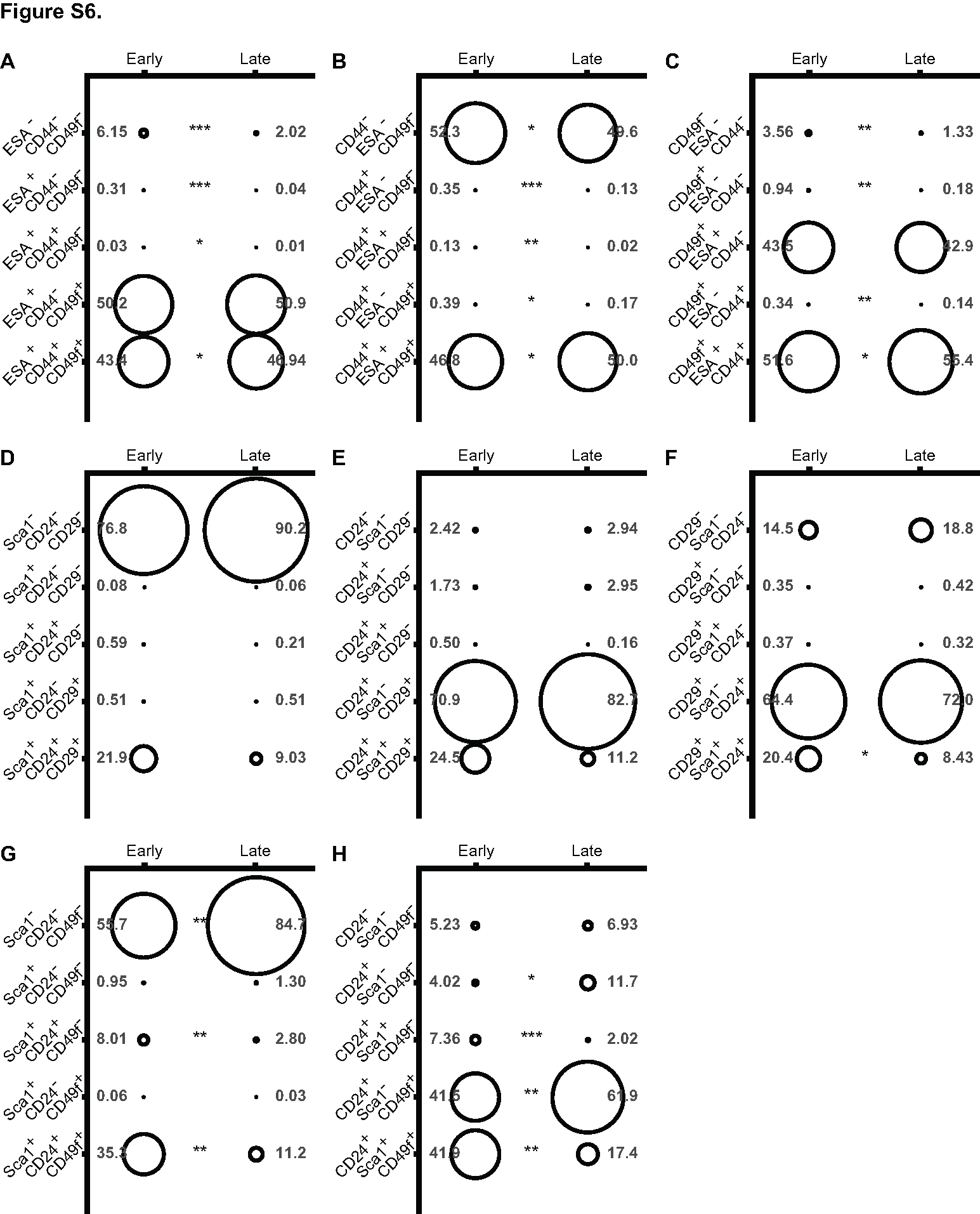

Supplement: Figure S6 — Multiparametric FACS analysis of cell surface markers expression in MMTV-Neu ME-CRCs. (TIFF) [file pone.0097666.s006.tif]
